# Glutamate Carrier Involvement in Mitochondrial Dysfunctioning in the Brain White Matter

**DOI:** 10.3389/fmolb.2020.00151

**Published:** 2020-07-21

**Authors:** Anne E. J. Hillen, Vivi M. Heine

**Affiliations:** ^1^Pediatric Neurology, Emma Children’s Hospital, Amsterdam UMC, Amsterdam Neuroscience, Vrije Universiteit Amsterdam, Amsterdam, Netherlands; ^2^Child and Youth Psychiatry, Emma Children’s Hospital, Amsterdam UMC, Amsterdam Neuroscience, Vrije Universiteit Amsterdam, Amsterdam, Netherlands; ^3^Department of Complex Trait Genetics, Center for Neurogenomics and Cognitive Research, Amsterdam Neuroscience, Vrije Universiteit Amsterdam, Amsterdam, Netherlands

**Keywords:** mitochondria, glutamate carriers, aspartate-glutamate carriers, white matter, leukodystrophy, excitotoxicity, cell type specificity, cell metabolism

## Abstract

Glutamate homeostasis is an important determinant of health of the central nervous system (CNS). Mitochondria play crucial roles in glutamate metabolism, especially in processes with a high energy demand such as action potential generation. Mitochondrial glutamate carriers (GCs) and aspartate-GCs (AGCs) regulate the transport of glutamate from the cytoplasm across the mitochondrial membrane, which is needed to control energy demand, lipid metabolism, and metabolic activity including oxidative phosphorylation and glycolysis. Dysfunction in these carriers are associated with seizures, spasticity, and/or myelin deficits, all of which are associated with inherited metabolic disorders. Since solute carrier functioning and associated processes are cell type- and context-specific, selective vulnerability to glutamate excitotoxicity and mitochondrial dysfunctioning is expected. Understanding this could offer important insights into the pathomechanisms of associated disorders. This perspective aims to explore the link between functions of both AGCs and GCs and their role in metabolic disorders, with a focus on a subclass of lysosomal storage disorders called leukodystrophies (LDs).

## A Role for Mitochondrial Glutamate Carriers in Inherited Metabolic Disorders

Mitochondrial dysfunction can lead to affected myelin metabolism (Carvalho, 2013), glutamatergic neurotransmission deficits (Vos et al., 2010), and excitotoxicity (Schinder et al., 1996; Wang and Thayer, 1996), and is associated with inherited metabolic disorders (Saffari et al., 2017). Mitochondria dynamically regulate energy homeostasis by controlling mitochondrial numbers (Ploumi et al., 2017) and by continuously relocating to subcellular locations with high energy demands (Alshaabi et al., 2019). They rely on active glutamate metabolism in order to partake in multiple metabolic pathways (Mahmoud et al., 2019). Glutamate is the most abundant amino acid and neurotransmitter in the central nervous system (CNS), and levels are meticulously regulated (Mahmoud et al., 2019). Excessive concentrations can cause excitotoxicity, leading to calcium influx, mitochondrial dysfunction, and subsequent cell death (Kritis et al., 2015). Glutamate homeostasis is regulated by cytoplasmic as well as mitochondrial carriers (Fiermonte et al., 2002; Berkich et al., 2007; Amoedo et al., 2016; Goubert et al., 2017; Mahmoud et al., 2019). Here, we will discuss the glutamate carriers (GCs) and aspartate-GCs (AGCs), which transport glutamate over the mitochondrial membrane, in the context of leukodystrophies (LDs), a subclass of lysosomal storage disorders. Considering the central role of astrocytes in glutamate metabolism, we will highlight mitochondrial defects in the astrocytopathies Alexander disease (AxD) and Vanishing White Matter (VWM) disease. These illustrate the close link between white matter pathology and astrocytic dysfunction, and will be used to look into how mitochondrial glutamate exchange might be coupled to cell type-specific pathology.

## Mitochondrial Aspartate-Glutamate Carriers

Aspartate-GC 1 [AGC1, SLC25A12, Aralar(1)] and 2 (AGC2, SLC25A13, Citrin) transport glutamate plus a proton into the mitochondrion in exchange for aspartate, and in this way support Malate-Aspartate Shuttle (MAS) activity. As cytosolic NADH cannot pass the inner mitochondrial membrane, NAD^+^ is transported instead together with malate, which is formed from aspartate that is transported by AGCs. Once inside the mitochondrial matrix, the NAD^+^ is reduced and the resulting NADH donates electrons to the mitochondrial electron transport chain, supporting ATP production (Satrustegui et al., 2007). Mitochondrial NADH levels are also increased due to additional NAD^+^ reduction upon conversion of malate to oxaloacetate, while the reverse takes place in the cytosol. Such AGC-mediated regulation of NADH/NAD^+^ ratios aids glycolysis, which requires cytosolic NAD^+^ molecules (Amoedo et al., 2016; Alkan et al., 2018). By transporting mitochondrial NH_3_-derived aspartate into the cytoplasm (Meijer et al., 1972), AGCs facilitate the urea cycle and associated NADH levels in the liver. In accordance, an increased cytosolic NADH/NAD^+^ ratio caused by AGC2 dysfunction disrupts urea cycle metabolism (Moriyama et al., 2006).

In neurons, aspartate that is transported into the cytoplasm by AGCs is converted into N-acetylaspartate (NAA) and shuttled to oligodendrocytes (Dahlin et al., 2015). In oligodendrocytes, NAA is metabolized into acetate, which in turn serves as a substrate for acetyl-CoA, forming fatty acids and ultimately myelin lipids (Dahlin et al., 2015). As AGCs play essential roles in regulating glutamate and aspartate homeostasis in mitochondrial and cytoplasmic compartments, they are involved in mitochondrial oxidative phosphorylation, lipid metabolism, the urea cycle, and glycolysis in the cytoplasm (Lasorsa et al., 2003; Amoedo et al., 2016).

In the CNS, AGC1 is the most highly expressed isoform (Iijima et al., 2001). AGC1 expression is high in neurons compared to oligodendrocytes (Profilo et al., 2017) and astrocytes (Ramos et al., 2003; McKenna et al., 2006; Berkich et al., 2007; Juaristi et al., 2019). While still debated, some report astrocytic AGC1 expression that increased with maturity (Li et al., 2012) and could support MAS function (Hertz, 2011). AGC1 is activated by an external regulatory binding site for calcium (Contreras et al., 2007; Satrustegui et al., 2007), stimulating the MAS and subsequent ATP production (Lasorsa et al., 2003; Gellerich et al., 2012). AGC2 is predominantly expressed in the liver, kidneys, and heart (Begum et al., 2002), and its neuronal expression is much more spatially restricted than AGC1s (Contreras et al., 2010; Profilo et al., 2017). Tissues expressing more AGC2 than AGC1 generally show high expression of genes involved in the urea cycle (Begum et al., 2002). The kinetics and calcium sensitivity of the two carriers also differ (Palmieri et al., 2001; Contreras et al., 2007). AGC2 dysfunction by mutations in the *SLC25A13* gene leads to citrullinemia type II (Kobayashi et al., 1999; Saheki et al., 2002). This disease primarily shows dysfunction in the liver where no AGC1 is present, suggesting some compensatory function between the two isoforms occurs when co-expressed. However, the role of AGC2 in the CNS has not been studied extensively and its exact function there remains elusive.

AGC1 deficiency is associated with epilepsy (Falk et al., 2014), hypomyelination (Wibom et al., 2009), and reduced NAA (Jalil et al., 2005; Falk et al., 2014; Profilo et al., 2017), which is an important substrate for myelin lipids. Furthermore, oligodendrocyte precursor cell (OPC)-specific AGC1 deficiency resulted in inhibited proliferation and increased maturation, suggesting an interactive pathway between AGC1 and oligodendrocytes in addition to the provision of NAA (Petralla et al., 2019).

## Mitochondrial Glutamate Carriers

Glutamate carrier 1 (GC1; SLC25A22) and 2 (GC2; SLC25A18) also transport glutamate over the mitochondrial inner membrane. The glutamate is again co-transported with a proton, allowing glutamate dehydrogenase inside the mitochondrial matrix to generate NH_3_ for ureogenesis (Meijer et al., 1972), and α-ketoglutarate to be used in the tricarboxylic acid (TCA) cycle (Hudson and Daniel, 1993; Li et al., 2017) or for gluconeogenesis (Stumvoll et al., 1997). Calcium signaling induces a preferential transamination of glutamate with oxaloacetate to form aspartate and subsequent aspartate efflux by AGCs, curtailing the input of AGCs to the TCA cycle (see Satrustegui et al., 2007) as compared to that of GCs. Different GC kinetics result in faster glutamate transport by GC1 than by GC2, suggesting a division of workload into basal and on-demand glutamate metabolism (Fiermonte et al., 2002). GC1 and GC2 are present in oligodendrocytes (Sun et al., 2015) and in a subset of neurons (Scifo et al., 2013; Llavero Hurtado et al., 2017). Studies also suggest a relatively high expression of GC1 and GC2 in astrocytes, as GC1 levels are higher in cortical astrocytes than in whole cortex-samples (Berkich et al., 2007) and GC2 is enriched in astrocyte endfeet containing an abundance of mitochondria (Mathiisen et al., 2010; Boulay et al., 2017). Others showed that GC2 expression is higher in the cortex than in the cerebellum and increases with age in both regions (Orre et al., 2014; Boulay et al., 2017; Boisvert et al., 2018). These data indicate GC expression levels differs based on neural cell type, brain region, and age, likely resulting in functional heterogeneity.

Glutamate carrier dysfunction is directly associated with epilepsy (Molinari et al., 2009; Poduri et al., 2013), among other neural defects. GC1 is an interaction partner of Battenin, encoded by neuronal ceroid lipofuscinosis-3 (CLN3; Scifo et al., 2013), which has been linked to intracellular trafficking and autophagy (Behrends et al., 2010). CLN3 mutations lead to neuronal ceroid lipofuscinosis (NCL; Luiro et al., 2006), a family of lysosomal storage diseases collectively referred to as Batten disease. Neural pathology presents with lipopigment deposits, and with mitochondrial dysfunction in interneurons in the cortex, thalamus, and hippocampus, and in cerebellar Purkinje cells (see Nelvagal et al., 2019). Primary NCL cortical microglia and astrocyte cultures show impaired glutamate clearing and desynchronized calcium waves (Parviainen et al., 2017). Experimental inactivation of GC1 in astrocytes resulted in decreased NAD^+^ and ATP levels, as well as an intracellular accumulation of glutamate that the authors proposed (Goubert et al., 2017) may lead to the altered neuronal synchronicity and epilepsy seen in GC1-deficient patients (Molinari et al., 2009). GC2 expression appears linked to inflammation levels, as GC2 levels significantly decreased upon spinal cord injury (Anderson et al., 2016) whereas increased GC2 expression was shown after inflammatory compound treatment of macrophage cultures (Hans et al., 2019). Because glial scar formation protects against inflammation (Sofroniew, 2015), GC2 could be involved in modulating inflammatory conditions. It is clear that GCs are crucial for glutamate metabolism and energy supply to the cell. Links to autophagy and inflammation also suggest a potential role for GC1 in white matter health and merit further studies in the context of clinical presentations.

## Mitochondrial Dysfunctioning and (A)GC Expression in White Matter Disorders

**FIGURE 1:**
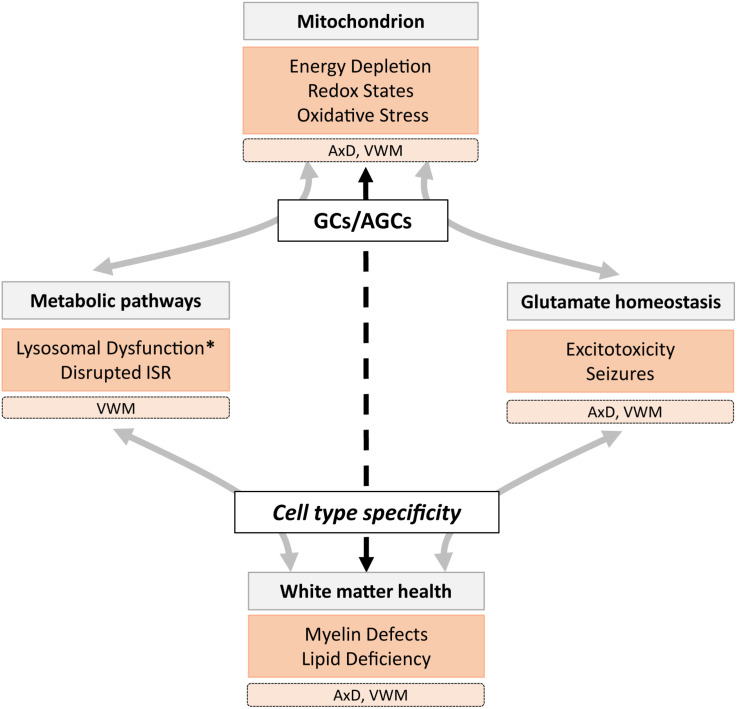
A schematic overview of the role of mitochondrial glutamate carriers GCs and AGCs in metabolic and glutamate homeostasis mechanisms involved in healthy (gray) and pathological (orange) states associated with lysosomal storage diseases (LSDs), including the leukodystrophies AxD and VWM. We hypothesize that (A)GC (dys)function has cell- and region-specific effects (dashed arrow) that affect white matter health directly and indirectly. Pathological states marked with an asterisk are not observed in AxD and VWM, but are present in many LSDs.

(A)GC deficiency is associated with hypomyelination (Wibom et al., 2009), epilepsy (Falk et al., 2014), spasticity (Molinari et al., 2005), and disruption of metabolic pathways, including oxidative phosphorylation, the urea cycle, and the MAS. These functions are regulated by different mechanisms, depending on cell type, brain region, and cellular state. First, neurons and astrocytes share similar machinery for glutamate metabolism but utilize it differently under basal conditions (Frigerio et al., 2008; Llorente-Folch et al., 2016). Second, glutamate levels in white matter areas are about half of that in gray matter areas (Hassel et al., 2003), caused by more effective glutamate-to-glutamine metabolism in white compared to gray matter astrocytes (Lundgaard et al., 2014). Third, cellular conditions such as stress, low energy supply, and altered calcium levels can affect intra- and extracellular glutamate levels. Thus, the relationship between mitochondrial metabolism and glutamate differs across cell types, is highly interactive, and supports various brain functions. It is therefore expected that certain neural structures and conditions show increased vulnerability to (A)GC dysfunction (Figure 1). We explore a role for (A)GCs in mitochondrial dysfunction in two LDs that present with symptoms similar to (A)GC deficiency. Before we discuss how cell types affected in these LDs might be vulnerable due to downstream effects of dysregulated (A)GC functioning, a short introduction into the mitochondrial symptoms of the two astrocytopathic LDs is given.

### Mitochondrial Dysfunctioning in Alexander Disease

Alexander disease is caused by mutations in the GFAP gene, which encodes for intermediate filaments in astrocytes. The AxD brain shows ultrastructurally abnormal mitochondria closely located to Rosenthal fibers, a hallmark of AxD pathology (Herndon et al., 1970; Caceres-Marzal et al., 2006), and shows an oxidative stress response (Castellani et al., 1998; Wang et al., 2011) in astrocytes of the white matter (Hagemann et al., 2006). Perturbed mitochondrial transfer was confirmed in models of AxD (Gao et al., 2019), and GFAP mutations were directly linked to reduced glial glutamate buffering (Wang et al., 2011). Interestingly, AxD patient iPSC-derived astrocytes showed downregulation of both GCs and both AGCs (Table 1), as well as less glycolysis, high oxidative respiration rates, lowered calcium release, and decreased extracellular ATP (Jones et al., 2018). Others showed that GFAP mutations are associated with decreased GLT-1 expression in astrocytes, concurrent with a decreased ability to rescue neurons from glutamate-induced excitotoxicity (Tian et al., 2010). These studies support the notion of secretion-related pathology of AxD astrocytes and involvement of affected glutamate homeostasis by mitochondrial dysfunctioning.

**TABLE 1:** Overview of findings of altered (A)GC expression in leukodystrophies.

Disease	Model and cell type	Technique	Expression	References
VWM	R132H VWM MEFs (mouse)	Mass spectrometry	Gel: up* *Agc1*: down∼ Agc2: n.s.	Raini et al., 2017
	R195H VWM Bergman glia (mouse)	RNA sequencing	*Gc1:* down* at 2, 5, and 7 m *Gc2:* n.s. at 2 m; down* at 5 m; down∼ at 7 m *Agc1:* down** at 2 and 5 m; down* at 7 m)	Wong et al., 2019
AxD	R88C AxD iPSC derived-astrocytes (human)	RNA sequencing	*GC1*^‡^: down, 1.2x *GC2*^‡^: down, 2.85x *AGC1*^‡^: down, 1.9x *AGC2*^‡^: no change, 1.0x	Jones et al., 2018
4H	M852V 4H oligodendrocyte cell line (human)	RNA sequencing	*GC1:* down* *AGC2:* down n.s.	Choquet et al., 2019
		Mass spectrometry	AGC2: down*	
Pelizaeus-Merzbacher disease	*Jimpy* OPCs/oligodendrocytes/astrocytes, during OPC differentiation (mouse)	RNA sequencing	*Gc2:* n.s/n.s./down***	Elitt et al., 2018

*p < 0.05; **p < 0.01; ***p < 0.001; ∼p ≤ 0.08); n.s., not significant. m, months. ^‡^Average TPM value fold change is used, with fold change calculated as control line divided by AxD line. (A)GC isoforms not mentioned were not found in the database.

### Mitochondrial Dysfunctioning in Vanishing White Matter Disease

Vanishing White Matter (VWM) disease is caused by mutations in the genes encoding for eukaryotic translation initiation factor EIF2B subunits. Astrocytes are primary affected by VWM mutations in both mice and patient tissues (Dooves et al., 2016). A hallmark of its white matter pathology is foamy oligodendrocytes with membranous vacuoles composed of mitochondrial membranes and myelin lamellae. They contain mitochondrial numbers of up to five times the amount in healthy appearing oligodendrocytes (Wong et al., 2000). Various models of VWM implicate mitochondrial and glutamate dysfunction. Raini et al. (2017) report that primary astrocytes obtained from VWM mice showed decreased oxidative phosphorylation, which was partially compensated by increased mitochondrial numbers and glycolysis. Primary VWM mouse embryonic fibroblasts (MEF) showed GC1 protein upregulation as well as a trend toward decreased AGC1 levels (Raini et al., 2017; Table 1). In severely affected VWM mice, a significant decrease in *Gc1* and *Agc1* transcripts was observed in the cerebellum (Wong et al., 2019; Table 1). Also, using VWM patient iPSCs, various differentially expressed mitochondrion-related genes genes were found in white matter astrocytes (Leferink et al., 2019). Reduced Sigma-1 receptor expression in VWM mouse astrocytes furthermore points to disrupted mitochondria-ER communication in VWM (Atzmon et al., 2018). Taken together, the mitochondrial dysfunction in VWM pathomechanisms suggests a role for (A)GCs.

## Mitochondrial Glutamate Carrier Involvement in White Matter Defects

### Deficient Mitochondrial Calcium Buffering Leads to Glutamate Excitotoxicity

Upon increased cytosolic calcium, mitochondrial carriers are activated to adjust glutamate supply to the mitochondria and to regulate ATP production via oxidative phosphorylation (Gellerich et al., 2012). High calcium levels within mitochondria lead to depolarization of the mitochondrial membrane (Duchen, 2000), increased production of radical oxygen species, and cell death (Orrenius et al., 2003). Because glutamate buffering in the brain is most pronounced in astrocytes (Danbolt et al., 2016), perturbed astrocytic glutamate uptake due to mitochondrial dysfunction contributes to a great degree to excitotoxic states and overall brain dysfunctioning. While the exact mechanism is unknown, GC1 dysfunction in astrocytes resulted in decreased ATP levels and accumulation of intracellular glutamate (Goubert et al., 2017), which can cause reversed glutamate transport into the extracellular space (Longuemare et al., 1999) and promote excitotoxicity. In neurons, it has been shown that glutamate-induced calcium buffering in mitochondria modulates NMDA glutamate receptor activity (Kannurpatti et al., 2000) and prevents excitotoxicity (Wang and Thayer, 1996). In astrocytic processes, mitochondria, AGC1, and GC1, are co-compartmentalized with cytosolic GLT-1 glutamate receptors (Genda et al., 2011; Ugbode et al., 2014), suggesting a coupling of energy demand of cytoplasmic glutamate transport and energy supply by mitochondrial GCs. Indeed, AxD presents with downregulated GLT-1 levels (Tian et al., 2010), alongside diminished calcium wave propagation and ATP export (Jones et al., 2018) and increased sensitivity to excitotoxicity and seizure development (Hagemann et al., 2012). The lineage of oligodendrocytic cells shows high vulnerability to glutamatergic excitotoxicity (Domercq et al., 2011), which could contribute to white matter damage in metabolic and lysosomal storage disorders. Glutamate excitotoxicity during white matter dysfunction is mainly studied in context of cytosolic GCs. However, considering the central role of (A)GCs in regulating calcium homeostasis upon glutamate level changes, mitochondrial carrier (dys)function should receive more attention.

### MAS Dysfunction Affects Oligodendrocyte Maturation and Myelin Production

Myelination requires mitochondrial production of acetyl-CoA, metabolized from neuronal aspartate (Dahlin et al., 2015), which is supported by oligodendrocyte lineage cell activity that increases mitochondrial ATP production and mitochondrial transcript levels (Silva et al., 2009; Schoenfeld et al., 2010). In line with this, AGC1 deficiency leads to decreased OPC proliferation (Petralla et al., 2019) and also to myelin deficits considering its function in the MAS (Wibom et al., 2009). The OPC maturation defects in VWM and AxD (Li et al., 2018; Leferink et al., 2019) could be associated to dysfunction of mitochondria, and specifically of AGC1 (Petralla et al., 2019). The MAS function of AGCs furthermore regulates the balance of glycolysis in the cytosol and oxidative phosphorylation in the mitochondrion (Lasorsa et al., 2003; Kasai et al., 2019). AGC1 expression is coupled to increased glycolysis (Lasorsa et al., 2003), increased glutamate oxidation (Herbst and Holloway, 2016), and is activated by calcium (Menga et al., 2015). These qualities of AGC1 suggest a link between neural and mitochondrial activity on the one hand, and oligodendrocyte differentiation and myelin metabolism on the other. Interestingly, both AxD and VWM astrocytes showed dysregulated oxidative phosphorylation, increased glycolysis and altered AGC1 expression (Raini et al., 2017; Jones et al., 2018), suggesting that these white matter disorders could share a common affected pathway. However, other neural subtypes could be affected as well, as a perturbed MAS is further determined by the different metabolic needs across cell types. Taken together, disrupted MAS activity likely plays a role in pathomechanisms of white matter deficiencies.

### Signaling Pathways Interact With Mitochondrial Functioning in a Cell Type-Dependent Manner

Of additional interest are metabolic pathways that interact with mitochondrial metabolism and that are regulated in a cell type-dependent manner. While the mitochondrial genome consists of only 37 genes, its transcription is modulated by many nuclear factors (Nunnari and Suomalainen, 2012). Conversely, mitochondrial dysfunction can regulate nuclear transcription (Suomalainen and Battersby, 2018), depending on cell type-specific differences (Bolea et al., 2019). Several mitochondrial genes contain cAMP response element (CRE) sequences. The associated transcription factor CRE-binding protein (CREB) regulates differential functional pathways in astrocytes and neurons, with more pronounced effects on mitochondrial metabolism in astrocytes (Pardo et al., 2017). The AGC1 promotor regions contain a CRE site, which in neurons leads to increased *AGC1* expression after binding of CREB upon its calcium-induced phosphorylation (Menga et al., 2015). CREB-dependent transcription additionally upregulates *Sigma-1 receptor* expression, and leads to lowered astrocytic excitability by decreasing ATP-dependent subcellular calcium waves (Eraso-Pichot et al., 2017). ATF4 is another CREB protein. It is part of the Integrated Stress Response (ISR) and is upregulated following mitochondrial disease or inhibition (Silva et al., 2009). An ATF4–CRE interaction is interesting with respect to VWM, considering the constitutive ISR activation in this disease (Abbink et al., 2019; Wong et al., 2019). In addition, *Sigma-1 receptor* expression is decreased in VWM (Atzmon et al., 2018). Interestingly, basal activation of the ISR in two forebrain astrocyte populations and in Bergmann glia of the cerebellum was significantly higher compared to other cell types (Wong et al., 2019), pointing toward an astrocyte-specific vulnerability to ISR disruption. Differential effects across cell types are further complicated by the interaction of ISR activation and mitochondrial functioning through Transcription factor EB (TFEB) and Transcription Factor A, Mitochondrial (TFAM). TFEB regulates autophagy and lysosomal biogenesis (Settembre et al., 2011) and is activated in response to ER stress and starvation (Martina et al., 2016; Yoneshima et al., 2016). Tfam knockout upregulated Tfeb and lowered NAD^+^ levels (Baixauli et al., 2015). It caused apoptosis in neurons (Beckervordersandforth et al., 2017) but not in astrocytes, although it did abolish their neuroprotective qualities (Fiebig et al., 2019). These data illustrate the complexity and importance of taking cell type-specific pathway regulation into account when investigating functional effects of (A)GCs.

## Conclusion

A prominent role for mitochondrial dysfunction and in particular mitochondrial glutamate pathways has been explored in inherited metabolic disorders characterized by white matter abnormalities. The role of (A)GCs in MAS function, (myelin) metabolism, and glutamate homeostasis, paired with the importance of astrocytes in glutamatergic homeostasis, align well with the central role of glial cells in many metabolic diseases. A more detailed interrogation of GC and AGC (in)activation, in various cell types within a single model, would be of interest in order to elucidate underlying disease mechanisms in white matter disorders.

## Author Contributions

AH and VH wrote the manuscript.

## Conflict of Interest

The authors declare that the research was conducted in the absence of any commercial or financial relationships that could be construed as a potential conflict of interest.
